# Influence of Ni Contents and Local Hydrogen Concentration on Crack Propagation in FCC Fe-Ni Alloy Models: A Molecular Dynamics Study

**DOI:** 10.3390/ma19153304

**Published:** 2026-08-04

**Authors:** Kaimeng Wang, Yingli Li, Molin Su, Hongqiao Yan, Yue Zhao, Lei Zhao

**Affiliations:** 1 CNPC Research Institute of Safety & Environment Technology, Beijing 102206, China; 2 School of Materials Science and Engineering, Tianjin University, Tianjin 300350, China

**Keywords:** Fe-Ni Alloy Models, crack propagation, hydrogen diffusion, molecular dynamics

## Abstract

This study investigates the atomic-scale effects of hydrogen concentration and Ni content on crack propagation in Fe-Ni alloy models using molecular dynamics methods. A Mode I crack model with a (001)[100] orientation was constructed, and hydrogen atoms were locally introduced at the crack tip with concentrations of 5.3 at.% and 14.3 at.%. Fe-Ni alloy models with 5%, 10%, 15%, and 20% Ni were compared in terms of crack growth, dislocation evolution, stacking fault energy, and hydrogen diffusion. The results show that local hydrogen introduction has a limited effect on the peak stress–strain response, while hydrogen clearly accelerates crack propagation in the middle stage, especially at high concentrations. For the 10% Ni model, the middle-stage crack growth rate increases to 0.36 Å/ps under 14.3 at.% crack-tip hydrogen. Crack growth in all models shows three stages. The 15% Ni model exhibits a clear plateau in the second stage and the shortest final crack length. Further analysis shows that Ni content regulates dislocation behavior through stacking fault energy. At 15% Ni, sustained dislocation entanglement and high-density dislocation multiplication occur near the crack tip, which helps dissipate local stress. Hydrogen diffusion analysis indicates that hydrogen mobility is lower in the 15% Ni model, which may be related to hydrogen retention near dislocation-rich regions. A normalized comparison based on hydrogen diffusion and middle-stage crack growth rate further identifies 15% Ni as the lowest crack propagation tendency composition among the studied models. These results provide atomic-scale data for Ni-content optimization in hydrogen-resistant alloys, although the direct engineering transfer of the findings is limited by the length and time scales of molecular dynamics simulations.

## 1. Introduction

As the global energy system moves toward cleaner energy sources, hydrogen has become an important energy carrier because of its clean combustion products and high energy density [1]. The large-scale use of hydrogen technologies depends on storage tanks, transmission pipelines, and other metallic components, which are usually made of iron-based alloys such as austenitic stainless steels and low-alloy pipeline steels [2,3]. Hydrogen damage is also common in oil and gas systems, where corrosive media such as H_2_S can produce atomic hydrogen that enters pipeline steels such as X52, X70, X80, and X100 steels [4,5,6,7]. Once hydrogen enters the steel matrix, it can interact with vacancies, dislocations, grain boundaries, and microcracks, leading to a loss of ductility, reduced toughness, and premature failure [8,9,10,11,12]. In severe cases, hydrogen can promote brittle fracture at stresses much lower than the yield strength [8,12,13,14,15]. Hydrogen-induced cracking has also been reported in pipeline steels exposed to hydrogen-containing environments, especially near weld joints or grain boundaries [4,16].

From an atomic scale perspective, hydrogen embrittlement (HE) results from the dynamic interaction between hydrogen atoms and microstructural defects. Several mechanisms have been proposed to explain this process. In a hydrogen-enhanced decohesion (HEDE) mechanism, hydrogen atoms segregate at grain boundaries or crack tips, weaken inter-atomic bonding, and reduce local inter-facial cohesion [17,18,19,20]. In a hydrogen-enhanced localized plasticity (HELP) mechanism, hydrogen promotes dislocation motion and leads to highly localized plastic deformation [15,20,21,22]. In a hydrogen-enhanced strain-induced vacancy formation (HESIV) mechanism, hydrogen accelerates the generation and clustering of strain-induced vacancies, which can further develop into nanovoids [13,23,24,25]. These mechanisms may operate together during crack initiation and propagation, making hydrogen-induced damage difficult to predict.

Experimental studies have provided important macroscopic information on crack propagation and damage evolution in hydrogen-containing environments. These data include crack growth rates, fracture toughness, and fracture morphology [26,27]. However, experiments still face limits in directly observing atomic-scale processes, such as hydrogen diffusion pathways and hydrogen–defect interactions. Atomic simulation methods can describe the interaction between hydrogen atoms and metallic microstructures at the atomic level. They provide support for understanding hydrogen-induced failure and hydrogen-resistant alloy design.

Atomic-scale studies have provided important insights into hydrogen diffusion, adsorption, trapping, and hydrogen–defect interactions in iron and steel. For hydrogen diffusion, Chen et al. [28] showed that a hydrogen diffusion coefficient at α-iron grain boundaries is much slower than that in bulk body-centered cubic (BCC) iron. Du et al. [29] found that tensile and compressive stresses can reduce hydrogen diffusion in α-iron. For hydrogen adsorption, Luo et al. [12] studied hydrogen adsorption on an Fe (110) surface and Li et al. [15] investigated hydrogen trapping and dynamic distribution in iron vacancies. These studies show that hydrogen transport and trapping are strongly affected by local defects and stress states.

Grain boundaries are important regions for hydrogen segregation, and their interaction with hydrogen can directly affect crack initiation and propagation. Jung et al. [17] investigated hydrogen-assisted grain-boundary crack propagation in BCC iron and showed that grain-boundary energy affects hydrogen segregation and crack resistance. Wang et al. [14] further showed that hydrogen and vacancies affect the tensile deformation behavior of Σ3 grain boundaries in pure Fe. Gou et al. [16] studied welded X80 steel and found that hydrogen-induced cracking is closely related to local microstructure and delayed dislocation emission. These studies indicate that hydrogen-assisted cracking is strongly controlled by local defect structures.

Dislocations and vacancies are major hydrogen trapping sites. Their interactions with hydrogen play a key role in plastic deformation and embrittlement. Yu et al. [22] showed that hydrogen-enhanced dislocation mobility can explain important features of the HELP mechanism. Xie et al. [10] showed that hydrogen atoms preferentially diffuse toward edge dislocation cores and form trapping configurations. Takai et al. [24] and Nagumo et al. [25] emphasized the important role of vacancies in hydrogen-related failure. Zhu et al. [21] investigated that hydrogen can change dislocation activity and twinning behavior depending on grain-boundary character and loading direction. These results show that hydrogen–dislocation and hydrogen–vacancy interactions are central to hydrogen-assisted deformation.

Precipitates and phase boundaries can also affect hydrogen embrittlement by trapping hydrogen or regulating dislocation motion. Wang et al. [30] found that hydrogen trapped at Cu precipitate and Fe matrix interfaces increases the critical resolved shear stress for dislocation motion. Qiao et al. [31] studied BCC and face-centered cubic (FCC) phase boundaries in X80 dual-phase pipeline steel, and identified a critical hydrogen concentration affecting phase transformation and dislocation activity, and local phase structure.

Atomic-scale simulations provide direct evidence for key hydrogen embrittlement mechanisms, including HELP, HESIV, and hydrogen-induced ductile-to-brittle transition, and enable partial quantitative predictions. Xing et al. [27] used molecular dynamics simulations to predict the critical hydrogen concentration for a ductile-to-brittle transition in α-iron. Wang et al. [32] showed that hydrogen distribution at crack tips in α-iron significantly affects cracking behavior. Zhou et al. [33] showed that hydrogen increases the cross-slip energy barrier in austenitic stainless steel, Fe_0.7_Ni_0.11_Cr_0.19_, which helps explain hydrogen-induced planar slip localization. Zhou et al. [18] further investigated grain-boundary fracture sensitivity in Fe_70_Ni_11_Cr_19_–1% H austenitic stainless steel. Early simulations by Song et al. [26] on pure iron also provided an important basis for atomic-scale studies of hydrogen embrittlement and hydrogen-induced crack propagation.

Although these studies have improved the understanding of hydrogen diffusion, trapping, segregation, and hydrogen–defect interactions, important questions remain for Fe-Ni alloys. Nickel plays a key role in stabilizing the microstructure of iron-based alloys. It affects austenite stability and precipitate formation, and it also strongly influences hydrogen solubility, hydrogen diffusivity, and hydrogen interactions with microstructural defects [34,35]. However, systematic studies on crack-tip hydrogen diffusion and crack propagation as a function of Ni content are still limited. In addition, most existing simulations use constant hydrogen concentrations. In real service environments, hydrogen levels may change with time and local conditions. Therefore, the coupled effects of hydrogen concentration and Ni content on crack-tip hydrogen accumulation, dislocation activity, and crack-path selection are still not fully understood.

Therefore, it is necessary to investigate crack propagation and crack-tip hydrogen diffusion in Fe-Ni alloys with different Ni contents and hydrogen concentrations. In this study, molecular dynamics simulations were used to examine the coupled effects of Ni content and crack-tip hydrogen concentration on Mode I crack propagation in Fe-Ni alloys. The main parameters considered in this work include crack growth behavior, dislocation nucleation and multiplication, stacking fault energy, and hydrogen diffusion near the crack tip. By comparing Fe-Ni models with different Ni contents, this work aims to clarify how Ni content affects hydrogen–dislocation interactions and crack propagation at the atomic scale.

## 2. Methods

**Figure 1:**
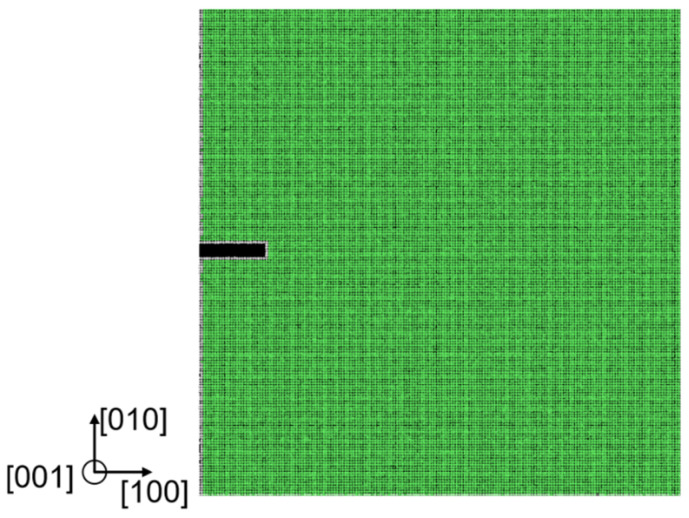
Coordinate axis orientation of Mode I crack propagation model. Green atoms represent FCC structures and white atoms represent other structures.

Mode I crack models of idealized FCC Fe-Ni alloy models were constructed using the LAMMPS (29 October 2020) simulation package. A typical model is shown in Figure 1, where free-surface boundary conditions were imposed along the x direction and periodic boundary conditions along the z direction. The crystallographic orientations of the simulation cell were set to [100], [010], and [001] along the x, y, and z axes, respectively. An initial crack with an orientation of (001)[100] was introduced on the left side of the simulation box, with a crack length of 4.2 nm and a width of 0.7 nm. A compressive pressure of 10 MPa was applied on the simulation box, followed by a relaxation period of 20 ps. Molecular dynamics simulations were performed in the canonical (NVT) ensemble [36], with the temperature maintained at 298.15 K (25 °C) using a Nose–Hoover thermostat [37,38]. A uniaxial tensile load was applied along the y direction at a strain rate of 1 × 10^9^ s^−1^ to open the pre-existing crack under Mode I-type tensile loading. In this work, the crack-tip driving force was not quantified by a stress intensity factor. Therefore, the crack propagation behavior was compared using the crack length evolution and the stage-wise crack propagation rate under the same loading condition.

Atomic interactions were described using an FeNiCrH embedded atom method (EAM) potential [18], which has been shown to accurately represent interactions among Fe, Ni, Cr, and hydrogen in Fe-Ni alloys. Although an FeNiCrH EAM potential was used, only the Fe-Fe, Fe-Ni, Ni-Ni, Fe-H, and Ni-H interactions were involved in the present Fe-Ni-H simulations. All simulations were performed using LAMMPS [39] with a timestep of 0.001 ps.

To simulate crack-tip propagation under hydrogen environments, hydrogen atoms were randomly inserted within the crack-tip region, followed by structural relaxation. Two local crack-tip hydrogen concentrations, 5.3 at.% and 14.3 at.%, were considered. These values refer to the hydrogen concentration in the selected crack-tip region rather than the bulk hydrogen concentration of the whole model.

In addition, FCC Fe-Ni alloy models with Ni contents of 5%, 10%, 15%, and 20% (denoted as A5, A10, A15, and A20) were constructed. Simulation trajectories were analyzed using the OVITO 3.8.2 visualization package [40]. The common neighbor analysis (CNA) method was employed to identify the local atomic structures, including FCC, hexagonal close packed (HCP), BCC, and other disordered structures. The dislocation extraction algorithm (DXA) was used to identify dislocation lines and dislocation types, including 1/6<112> Shockley partial dislocations and 1/3<100> Hirth dislocations. The evolution of dislocation density was obtained from the total dislocation line length divided by the model volume.

Crack propagation length was measured from the increase in crack length relative to the initial crack length during loading. The crack-tip atom was identified in OVITO, and its particle identifier was recorded. The atomic coordinates corresponding to this particle identifier were then extracted from the LAMMPS trajectory files using a Python 3.11.0 script. The crack-tip position was defined by the x-coordinate of the identified atom, because crack propagation occurred along the x direction in the present model. The crack propagation length was calculated based on the coordinate difference between the current and initial crack tips. The same procedure was applied to all models and all selected timesteps. The stage-wise crack propagation rate was obtained from the slope of the crack propagation length–time curve by linear fitting within each stage.

Atomic coordinates and the mean squared displacement (MSD) of hydrogen atoms were recorded during the loading process. The hydrogen diffusivity near the crack tip was usually evaluated by calculating the MSD of hydrogen atoms using LAMMPS implementation of the mean squared displacement, defined as:

(1) $$MSD(t)=\langle {\left|r(t)-r(0)\right|}^{2}\rangle$$

where the angle brackets denote averaging over all hydrogen atoms in the group. Because the MSD was calculated during active tensile loading, the obtained coefficient should be regarded as an apparent hydrogen mobility coefficient under dynamic deformation rather than an equilibrium diffusion coefficient.

The apparent hydrogen mobility coefficient D_app_ was then calculated from the MSD curve according to:

(2) $$D_{app}=\frac{1}{6}\frac{d}{dt}MSD(t)=\frac{1}{6}\frac{d}{dt}\langle {\left|r(t)-r(0)\right|}^{2}\rangle$$

## 3. Results

### 3.1. Crack Models with Different Hydrogen Concentrations at the Crack Tip

**Figure 2:**
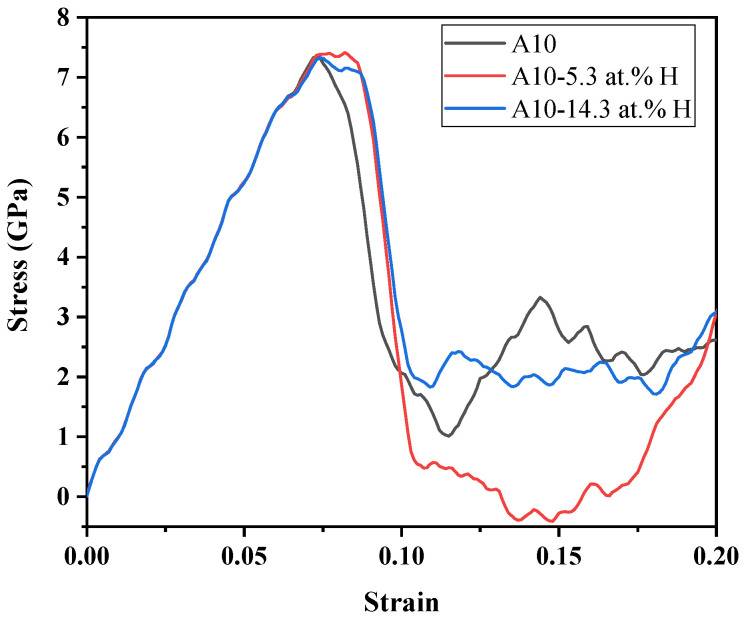
Stress–strain curves of the 10% Ni crack model with different crack-tip hydrogen concentrations.

For the (001)[100] oriented crack model, the A10 model (10% Ni) was used as a representative case. Figure 2 compares the stress–strain curves under three conditions: hydrogen-free, 5.3 at.% H at the crack tip, and 14.3 at.% H at the crack tip. The peak stresses of the 5.3 at.% H and 14.3 at.% H models are nearly identical, both close to 7.35 GPa. This is because hydrogen atoms were inserted only near the crack-tip region and did not significantly alter the overall atomic bonding energy of the system. Therefore, the yielding behavior and peak stress are mainly governed by the intrinsic mechanical properties of the FCC Fe-Ni alloy model matrix. The localized hydrogen effect is insufficient to cause a notable change in the peak stress.

**Figure 3:**
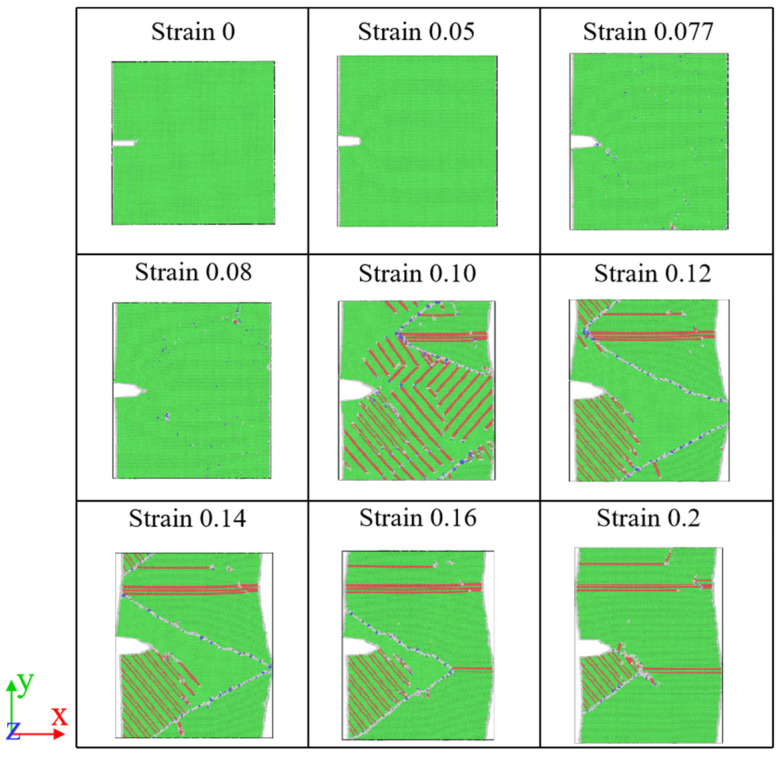
Atomic snapshots of Mode I crack propagation for the A10 model. Atomic structures were identified by CNA. Green atoms represent FCC structures, blue atoms represent BCC structures, red atoms represent HCP structures, and white atoms represent other structures.

**Figure 4:**
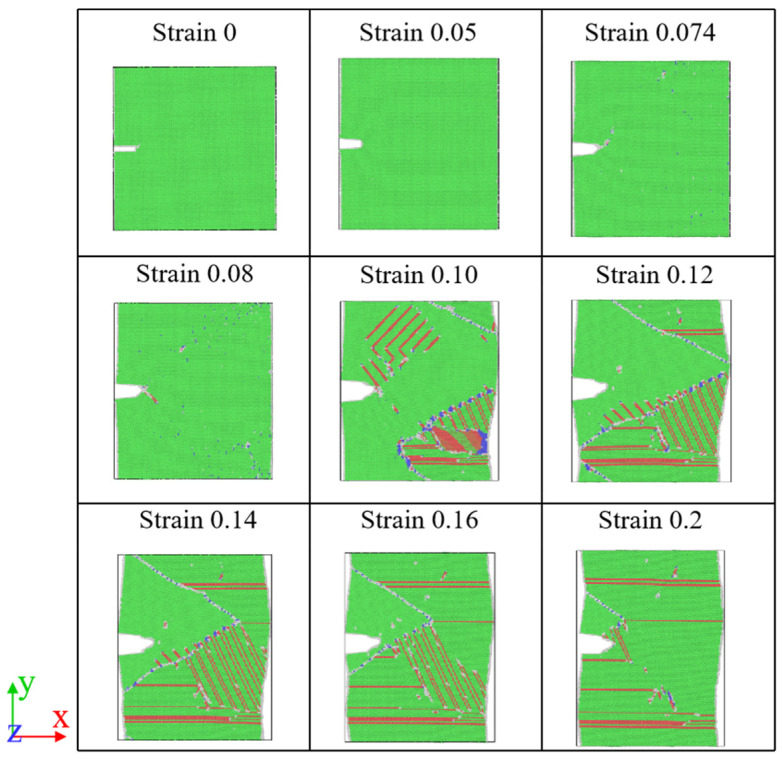
Atomic snapshots of Mode I crack propagation for the A10-5.3 at.% H model. Atomic structures were identified by CNA. Green atoms represent FCC structures, blue atoms represent BCC structures, red atoms represent HCP structures, and white atoms represent other structures.

**Figure 5:**
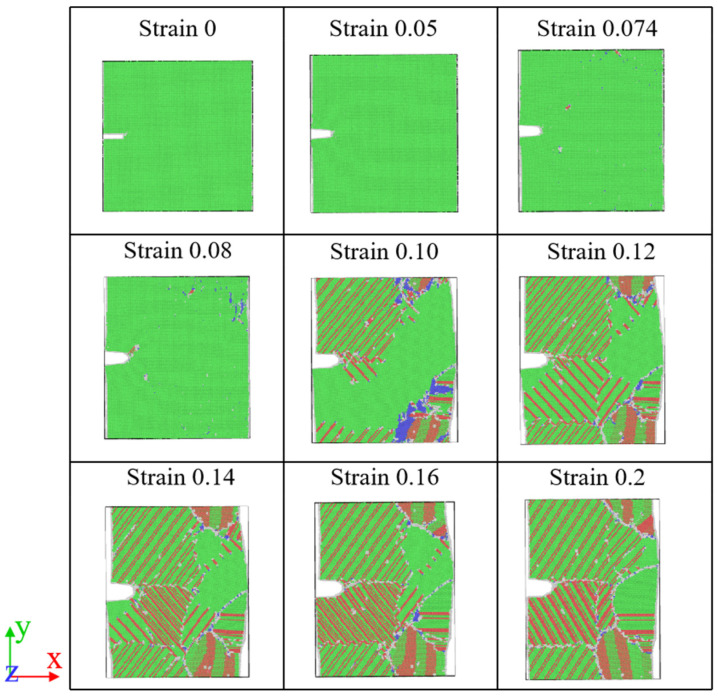
Atomic snapshots of Mode I crack propagation for the A10-14.3 at.% H model. Atomic structures were identified by CNA. Green atoms represent FCC structures, blue atoms represent BCC structures, red atoms represent HCP structures, and white atoms represent other structures.

Similarly, atomic snapshots of Mode I crack propagation for the A10 model with hydrogen-free, 5.3 at. %, and 14.3 at. % crack-tip hydrogen concentrations are shown in Figure 3, Figure 4 and Figure 5. In these figures, green atoms represent FCC structures, blue atoms represent BCC structures, red atoms represent HCP structures, and white atoms correspond to other structural states. Combining the atomic snapshots (Figure 3, Figure 4 and Figure 5) with stress evolution reveals that the crack propagation behavior is consistent across the different hydrogen concentrations.

When the strain is below 0.05, the atomic structure remains similar to that of the hydrogen-free model. At a strain of approximately 0.074, lattice disorder occurs at the crack tip due to stress concentration, and the stress reaches its peak value of 7.35 GPa. When the strain exceeds 0.08, HCP structures begin to appear at the crack tip, accompanied by an increase in stacking faults. Phase transformation also occurs at the corners of the simulation box. Although this phase transformation does not effectively suppress crack propagation, resulting in a gradual stress decrease, continued deformation leads to further widening of the transformed region, as well as pronounced increases in stacking fault density and crack size (both length and width).

**Figure 6:**
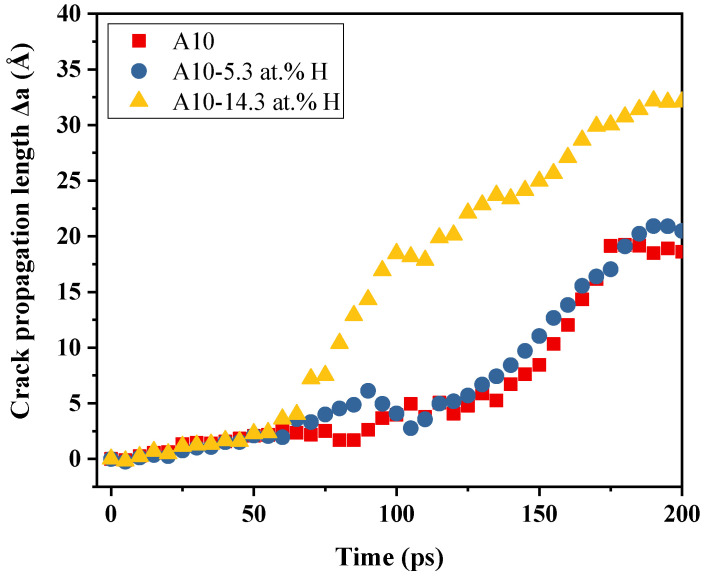
Crack propagation length versus time for the 10% Ni crack model with different crack tip hydrogen concentrations.

According to the relationships between crack propagation length and time for the models with different hydrogen concentrations, as shown in Figure 6, the crack propagation behavior exhibits three-stage characteristics: Stage I during 0–60 ps, Stage II during 60–110 ps, and Stage III during 110–200 ps. The crack propagation rates extracted from these stages are calculated in Table 1. The Stage I crack propagation rates show no significant difference among the three hydrogen concentrations. At the initial stage of crack propagation, the initial stress concentration at the crack tip is limited, resulting in an insufficient driving force for propagation. While in Stage II, the crack propagation rate increases noticeably with the increasing hydrogen concentration at the crack tip. In particular, the model with 14.3 at.% H exhibits a much higher crack propagation rate than the 5.3 at.% H model during this stage. The promotion effect of low concentration hydrogen on crack propagation is significantly weaker than that of high concentration hydrogen. High concentration hydrogen promotes dislocation motion at the crack tip, further accelerating crack growth.

In Stage III, the crack propagation rates of the A10 model and A10-5.3 at.% H model are identical, and both are significantly higher than those in Stage II. This phenomenon is attributed to the remarkable elevation of the stress intensity factor with the increase in crack length, where the stress intensity factor serves as the core mechanical driving force governing crack tip propagation. In contrast, the propagation rate of the A10-14.3 at. % H model in Stage III is lower than that in Stage II. When the crack propagates to a certain length, the stress concentration at the crack tip decreases, which weakens the mechanical driving force for further crack propagation.

### 3.2. Crack Models with Different Ni Contents

**Figure 7:**
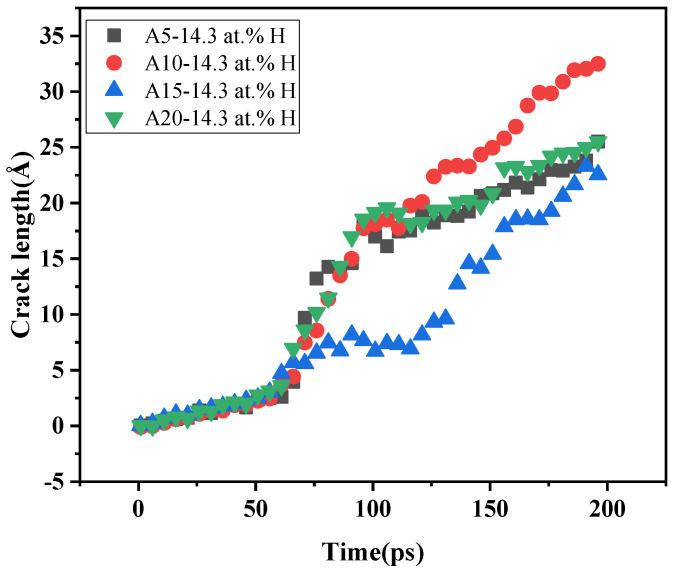
Time evolution of crack propagation length for FCC Fe-Ni alloy models with different Ni contents (A5, A10, A15, and A20) under the same local crack-tip hydrogen concentration of 14.3 at.% H.

The crack propagation behavior of the FCC Fe-Ni alloy model with different Ni contents also exhibits a three-stage evolution, as shown in Figure 7 (Stage I: 0–60 ps, Stage II: 60–110 ps, and Stage III: 110–200 ps). In this section, the comparison among A5, A10, A15, and A20 was conducted under the same local crack-tip hydrogen concentration of 14.3 at.% H. However, the A15 model shows a distinct deviation from this general trend.

During Stage I (0–60 ps), the Mode I crack propagation rates of all the models are similar, with crack propagation lengths within 5 Å. At this early stage, hydrogen aggregation does not induce significant differences among the samples.

During Stage II (60–110 ps), rapid crack propagation behavior is observed in the A5, A10, and A20 samples. In contrast, the A15 model exhibits a unique plateau region, in which the crack length remains nearly constant. This behavior differs markedly from the other compositions and suggests that the hydrogen–dislocation interaction at the crack tip of A15 in this stage maintains a temporary balance between the driving force and the resistance of crack propagation.

During Stage III (110–200 ps), the final crack lengths at 200 ps differ significantly across the models. The A10 model reaches the largest crack length (≈32 Å). The A5 and A20 models reach final crack lengths of approximately 18 Å and 25 Å, respectively, whereas the A15 model exhibits the smallest final crack length (≈22 Å). This indicates that the plateau region observed in Stage II of A15 model contributes to the reduced crack propagation over the entire deformation process.

The influence of Ni content on crack evolution can be attributed to its effect on the microstructural characteristics of an FCC Fe-Ni alloy, such as stacking fault energy and grain boundary structure, which in turn govern hydrogen diffusion and accumulation [41,42]. Under the same local crack-tip hydrogen concentrations, the reduced final crack length of the A15 model suggests that hydrogen may undergo a more balanced trapping and release process in this composition. This behavior may reduce the local driving force for crack propagation, manifesting as the observed plateau in Stage II.

## 4. Discussion

### 4.1. Stacking Fault Energy Calculation

Stacking fault energy (SFE) is a fundamental intrinsic parameter governing dislocation behavior in metallic materials. From the perspective of dislocation dynamics, SFE determines the dislocation core structure and associated deformation mechanisms [43,44]. At low SFE, perfect dislocations tend to dissociate into widely separated partial dislocations, leading to extended dislocation configurations. In this regime, cross-slips and climb are strongly suppressed due to the large separation between partials, confining dislocation motion to specific slip planes and promoting planar-slip behavior. Conversely, as the SFE increases, the dislocation core width becomes narrower, reducing the energetic barriers for cross-slips and climbs. Dislocations can therefore activate multiple slip systems, rearrange more flexibly, and facilitate stress relaxation through more efficient dislocation interactions.

Stacking fault energy was calculated using a separate FCC Fe-Ni cell with the same Ni content as the crack model. The simulation cell was oriented to expose the close-packed stacking sequence of the FCC structure. The cell was divided into bottom, middle, and top regions along the stacking direction. To generate an intrinsic stacking fault, one close-packed atomic layer in the middle region was removed, and the upper region was displaced along the stacking direction to close the gap and change the local stacking sequence. The structure was then relaxed by energy minimization. Stacking fault energy was calculated as

(3) $$\gamma_{SFE}=\left(E_{SF}-E_{0}\right)/A$$

where $E_{SF}$ is the total energy of the relaxed cell containing the stacking fault, $E_{0}$ is the total energy of the corresponding perfect FCC cell, and A is the stacking fault area.

**Figure 8:**
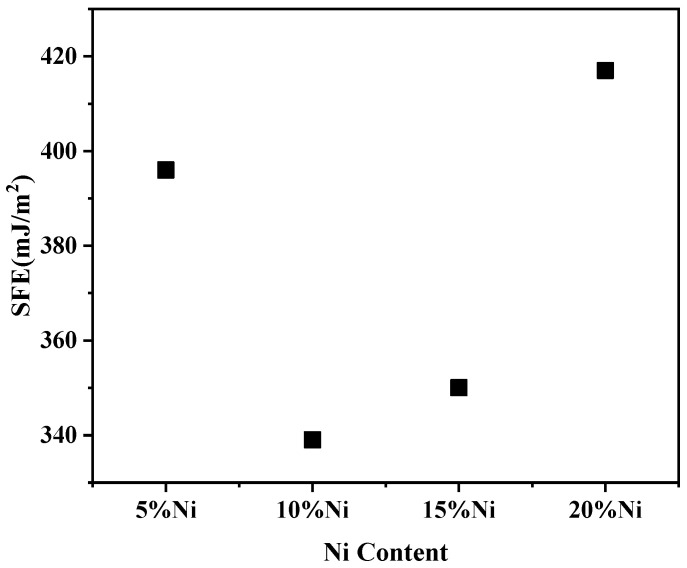
SFE of FCC Fe-Ni alloy models containing different Ni contents.

As shown in Figure 8, the calculated SFE values vary with Ni content in a non-monotonic manner in the present FCC Fe-Ni models. The A10 model shows the lowest SFE at 339 mJ/m^2^, followed by A15 at 350 mJ/m^2^. The A5 model has a higher SFE of 396 mJ/m^2^, while the highest SFE is observed in A20, reaching 417 mJ/m^2^. The calculated SFE values are higher than many reported values for commercial alloys. This difference may be related to the idealized FCC Fe-Ni model, the absence of additional alloying elements such as Cr, Mn, and N, the selected EAM potential, and the finite atomistic calculation conditions. Therefore, the absolute SFE values should not be directly compared with the experimental values of engineering alloys. In this work, these values are used mainly for relative comparison among the present models calculated using the same potential and the same SFE calculation procedure.

### 4.2. Dislocation Analysis

**Figure 9:**
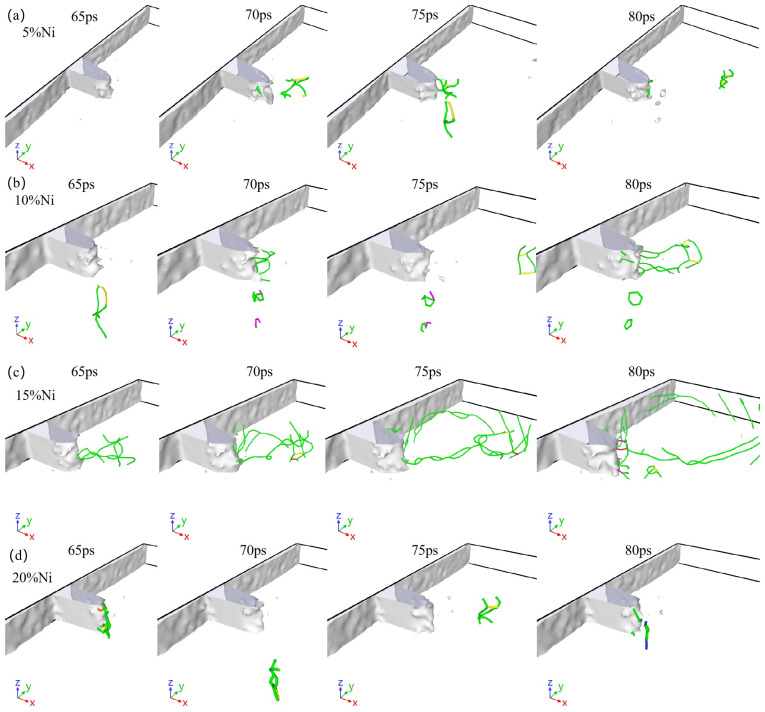
Dislocation evolution between 65 and 80 ps for models with different Ni contents under the same local crack-tip hydrogen concentration of 14.3 at.% H: (**a**) 5% Ni, (**b**) 10% Ni, (**c**) 15% Ni, and (**d**) 20% Ni. Dislocation lines were identified by DXA in OVITO. Green lines represent 1/6<112> Shockley partial dislocations, yellow lines represent 1/3<100> Hirth dislocations, blue lines represent 1/2<110> perfect dislocations, and 1/6<110> pink lines represent stair-rod dislocations.

The atomistic evolution of dislocation multiplication exhibits pronounced differences among FCC Fe-Ni alloy models with varying Ni contents, as shown in Figure 9. In the A5 model, no obvious dislocation accumulation is observed at the crack tip at 65 ps. At 70 ps, a small number of 1/6<112> Shockley dislocations appear ahead of the crack tip, but these dislocations are short and spatially dispersed. At 75 ps, the dislocation activity increases slightly, and several dislocation segments extend away from the crack-tip region. At 80 ps, the dislocation activity near the crack tip weakens again, and only a few short dislocation segments remain. This intermittent dislocation behavior indicates that the A5 model cannot maintain sustained dislocation multiplication near the crack tip during the second stage of crack propagation.

For the A10 model, no dislocations are observed at the crack tip at 65 ps, whereas ring-shaped configurations composed of 1/6<112> Shockley dislocations and 1/3<100> Hirth dislocations appear away from the crack-tip region. At 70 ps, stress concentration at the crack tip triggers dislocation emission, producing nine 1/6<112> Shockley dislocations. When at 75 ps, these crack-tip dislocations vanish, likely due to cross-slips followed by the annihilation of positive and negative dislocations, leaving only a small number of dislocations in regions farther from the crack tip. At 80 ps, another burst of dislocation multiplication occurs, generating sixteen 1/6<112> Shockley dislocations and two 1/3<100> Hirth dislocations at the crack tip, with two additional 1/6<112> dislocation loops remaining in the distant region.

In contrast, the A15 model exhibits sustained dislocation entanglement. At 65 ps, the crack tip emits fifteen 1/6<112> Shockley dislocations, which rapidly develop three-dimensional entanglement. With increasing time, the emitted dislocations extend in length while maintaining the tangled configuration. When at 75 ps, strong interactions among multiple dislocations form a braided structure, which is an intertwined dislocation structure, sliding along the (1$\bar{1}$1) plane. At 80 ps, additional dislocations continue to accumulate at the crack tip.

In the A20 model, eighteen short dislocations appear at the crack tip at 65 ps. When at 70 ps, crack-tip dislocations disappear, while a dislocation loop forms approximately 0.5 nm away from the crack tip. At 75 ps, the crack-tip region remains free of dislocation accumulation, and at 80 ps, only two 1/6<112> Shockley dislocations remain.

Combining these observations with the SFE results provides a mechanistic link between Ni content and dislocation behavior near the crack tip. In the A20 model, the high SFE produces narrow dislocation cores and significantly reduces the energy barrier for dislocation nucleation. Consequently, rapid emission of short dislocations is observed at 65 ps, confirming the strong promoting effect of high SFE on initial dislocation nucleation. However, because high SFE also lowers cross-slip barriers, dislocations readily escape the crack tip region through unrestricted cross-slip, causing dislocation density at the crack tip to drop sharply under 70 ps. The high escape rate relative to the multiplication rate suppresses sustained dislocation accumulation.

In the A10 model, the low SFE stabilizes widely dissociated extended dislocations, which preferentially glide on a single slip plane. At 65 ps, no localized crack-tip dislocations are observed because the nucleation barrier for extended partials is high. The ring-shaped extended dislocation configurations away from the crack tip reflect the planar nucleation characteristic of low SFE materials.

In the A15 model, the intermediate SFE provides an optimal balance between nucleation and cross-slip. At 65 ps, fifteen 1/6<112> Shockley dislocations are emitted from the crack tip. The moderate SFE reduces the barrier for cross-slip, while still limiting complete dislocation escape. This allows the dislocation motion to change from a planar glide to a three-dimensional cross-slip, which promotes the formation of initial dislocation entanglement. As loading continues, multiple slip systems are activated, and the dislocation structure evolves into a three-dimensional network. This evolution is reflected by the twisted, rope-like dislocation bundles moving along the (1$\bar{1}$1) plane at 75 ps. When at 80 ps, dislocation multiplication remains active, showing a sustained cross-slip entanglement multiplication mode. This behavior enhances crack-tip stress dissipation and contributes to the improved resistance to crack propagation in the A15 model.

A higher SFE generally narrows the separation distance between partial dislocations and reduces the resistance to cross-slip. This can promote dislocation glide and help release local stress at the crack tip. However, if the SFE is too high, dislocations can move away from the crack-tip region more easily, and sustained dislocation entanglement becomes difficult to maintain. In this case, the dislocation structure provides fewer stable trapping sites for hydrogen atoms. Although A5 and A20 have different Ni contents, their relatively high SFE values may reduce the stability of extended dislocation structures and make dislocation retention near the crack tip less favorable. As a result, both models show limited sustained dislocation entanglement compared with A15.

Overall, the four Ni-containing models show different dislocation evolution modes near the crack tip. The A5 and A10 models exhibit intermittent dislocation activity and limited sustained dislocation accumulation. The A20 model shows rapid dislocation emission in the early stage, but the dislocations do not remain near the crack tip for a long time. In contrast, the A15 model maintains continuous dislocation multiplication and entanglement during the second stage of crack propagation. This behavior may help dissipate local stress and is consistent with the crack growth plateau observed in the A15 model.

**Figure 10:**
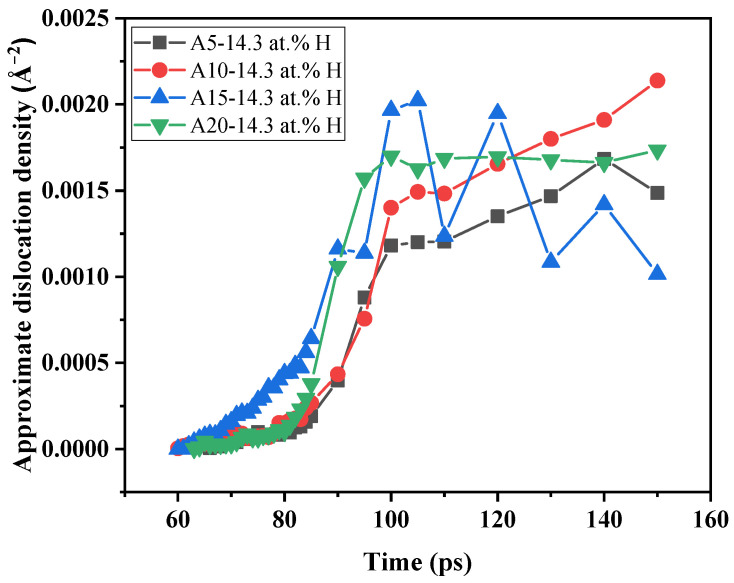
Evolution of approximate dislocation density with time for A5, A10, A15, and A20 models under the same local crack-tip hydrogen concentration of 14.3 at.% H.

Figure 10 shows the evolution of approximate dislocation density versus time for the different Ni content models. All four models (A5, A10, A15, and A20) exhibit rapid initial increases followed by slower fluctuations or a plateau-like stage. In the A5 model, the dislocation density remains low before 85 ps and then increases rapidly to about 0.0012 Å^−2^ near 100 ps. After that, it increases slowly and fluctuates within a moderate range, indicating limited sustained dislocation accumulation compared with A15. In the A10 model, the dislocation density increases continuously and reaches the highest value among the four models near 150 ps, suggesting delayed dislocation accumulation during the later propagation stage. In the A15 model, the dislocation density increases earlier and reaches a high transient value of about 0.0020 Å^−2^ around 100–105 ps, followed by downward fluctuations. This trend is consistent with the DXA observation of sustained crack-tip dislocation entanglement and may contribute to the crack growth plateau during Stage II. In the A20 model, the dislocation density increases rapidly before 100 ps and then remains at about 0.0017 Å^−2^. Compared with A15, both A5 and A20 show weaker sustained crack-tip dislocation accumulation. Therefore, the dislocation-density evolution supports that the A15 model has a more favorable balance between dislocation multiplication and crack-tip dislocation retention under the present simulation conditions.

### 4.3. Hydrogen Diffusion Analysis

**Figure 11:**
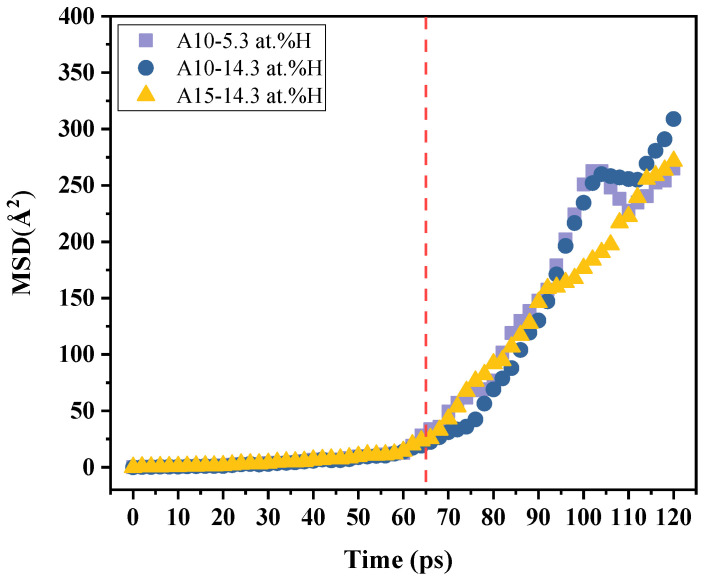
MSD evolution with simulation time.

Figure 11 shows the MSD of hydrogen atoms as a function of time. The MSD curves exhibit two distinct stages, and the apparent hydrogen mobility coefficients for each stage, which were calculated using Equation (2), are summarized in Table 2. In Stage I, the MSD increases slowly, indicating a relatively low apparent hydrogen mobility coefficient. Furthermore, at low hydrogen concentrations, the diffusion coefficient is significantly lower than that at high concentrations. In Stage II, the A15-14.3 at.% H model exhibits the lowest apparent hydrogen mobility coefficient, suggesting that the increased Ni content suppresses hydrogen mobility to some extent.

**Figure 12:**
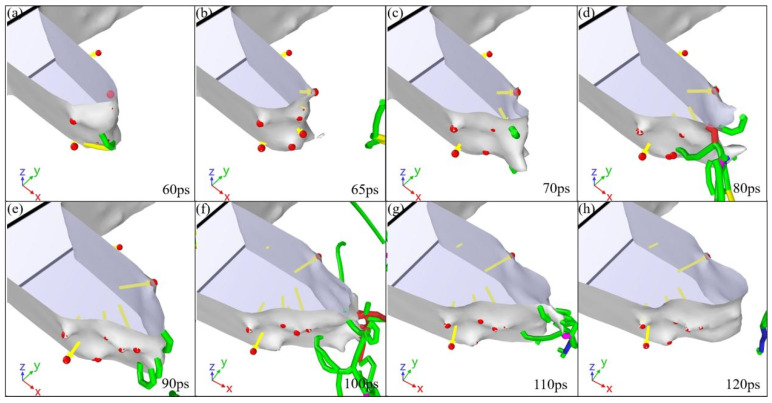
Atomic snapshot images of A10-5.3 at. % H model at different time steps: (**a**) 60 ps, (**b**) 65 ps, (**c**) 70 ps, (**d**) 80 ps, (**e**) 90 ps, (**f**) 100 ps, (**g**) 110 ps and (**h**) 120 ps, where red spheres represent hydrogen atoms and yellow arrows indicate displacement vectors. Dislocation lines were identified by DXA in OVITO. Green lines represent 1/6<112> Shockley partial dislocations, blue lines represent 1/2<110> perfect dislocations, yellow lines represent 1/3<100> Hirth dislocations, 1/6<110> pink lines represent stair-rod dislocations, and red lines represent other dislocations.

**Figure 13:**
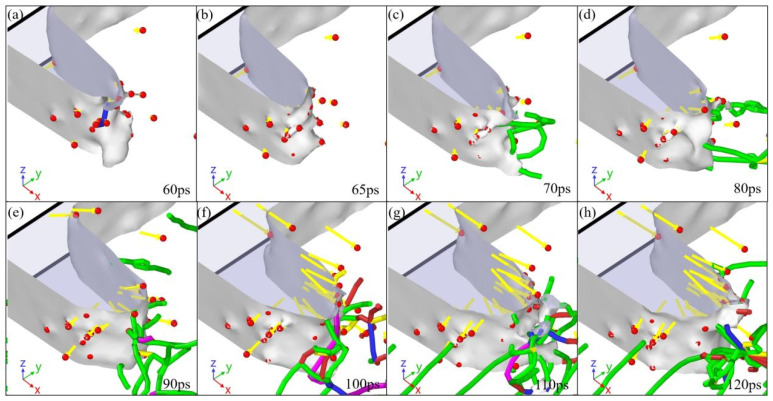
Atomic snapshot images of A10-14.3 at. % H model at different time steps: (**a**) 60 ps, (**b**) 65 ps, (**c**) 70 ps, (**d**) 80 ps, (**e**) 90 ps, (**f**) 100 ps, (**g**) 110 ps and (**h**) 120 ps, where red spheres represent hydrogen atoms and yellow arrows indicate displacement vectors. Dislocation lines were identified by DXA in OVITO. Green lines represent 1/6<112> Shockley partial dislocations, blue lines represent 1/2<110> perfect dislocations, 1/6<110> pink lines represent stair-rod dislocations, and red lines represent other dislocations.

Figure 12 and Figure 13 show the spatial distribution and migration of hydrogen atoms in the A10-5.3 at.% H and A10-14.3 at.% H models, respectively, at different simulation times. A comparison of the two models reveals that hydrogen atoms (red spheres) are more concentrated at the crack tip in the high-concentration model (A10-14.3 at.% H). At 100 ps and 120 ps, intense dislocation entanglement occurs near the crack tip, indicating that the elevated hydrogen concentration enhances dislocation multiplication by lowering the local dislocation nucleation barrier through hydrogen-induced stress field modification. In addition, the displacement vectors (yellow arrows) in the A10-14.3 at.% H model show broader dispersion and more diverse orientations, demonstrating more active hydrogen migration and more frequent interactions between hydrogen atoms and dislocations at high hydrogen concentrations.

**Figure 14:**
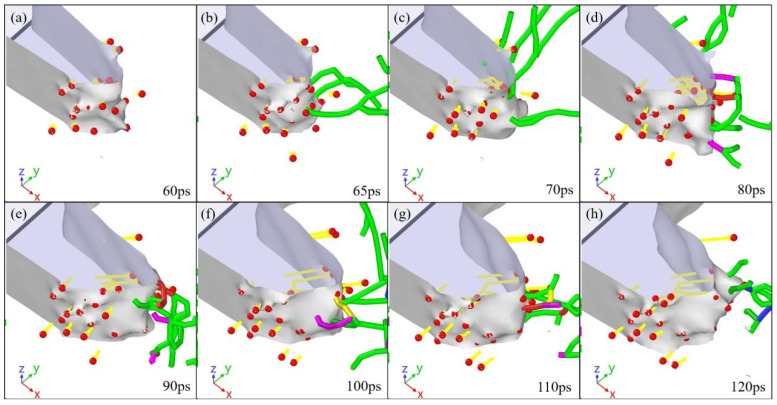
Atomic snapshot images of A15-14.3 at. % H model at different time steps: (**a**) 60 ps, (**b**) 65 ps, (**c**) 70 ps, (**d**) 80 ps, (**e**) 90 ps, (**f**) 100 ps, (**g**) 110 ps and (**h**) 120 ps, where red spheres represent hydrogen atoms and yellow arrows indicate displacement vectors. Dislocation lines were identified by DXA in OVITO. Green lines represent 1/6<112> Shockley partial dislocations, blue lines represent 1/2<110> perfect dislocations, 1/6<110> pink lines represent stair-rod dislocations, and red lines represent other dislocations.

Figure 14 presents the hydrogen distribution and dislocation evolution in the A15-14.3 at.% H model. Comparing Figure 13 and Figure 14, the A15 model already exhibits substantial dislocation entanglement at 65 ps and 70 ps, earlier than in the A10-14.3 at. % H model. This behavior, previously explained in Section 4.2, arises from the intermediate SFE of the A15 model, which promotes cross-slips and facilitates rapid three-dimensional dislocation interactions. When combined with hydrogen distribution, the displacement vectors in the A15-14.3 at.% H model show a reduced magnitude and lower dispersion. This indicates that at 15% Ni, the higher SFE narrows the dislocation cores and may enhance hydrogen retention near dislocation-rich regions, causing hydrogen atoms to be more easily trapped by dislocations and suppressing their diffusion.

Overall, the strong spatial overlap between dislocation multiplication zones and hydrogen-rich regions indicates a dynamic, mutually coupled interaction between hydrogen and dislocations. On the one hand, hydrogen promotes dislocation nucleation through its local stress field. On the other hand, SFE with different Ni contents modifies the dislocation core width and enhances hydrogen trapping, thereby restricting hydrogen diffusion.

### 4.4. Normalized Comparison of Hydrogen-Assisted Crack Propagation Resistance

To provide a clearer comparison among the four Ni-containing models, a simple normalized index was used as a supplementary evaluation. The normalized index was used only as a descriptive supplementary metric to combine two relative indicators: apparent hydrogen mobility and middle-stage crack propagation rate. It is not intended to represent a universal fracture-mechanics parameter. For each Ni content, the apparent hydrogen mobility coefficient $D_{H,\;i}$ and the crack propagation rate in the middle stage $v_{i}$ were normalized by the average values of the four models, expressed as ${\bar{D}}_{H,\;i}=D_{H,\;i}/D_{H,\;avg}$ and ${\bar{v}}_{i}=v_{i}/v_{avg}$. A normalized crack resistance index was then defined as $I_{i}=1/\left({\bar{D}}_{H,\;i}{\bar{v}}_{i}\right)$. A larger $I_{i}$ indicates a lower apparent hydrogen mobility coefficient and a lower crack propagation rate relative to the average level. Because this index can be sensitive to small values of $D_{H,\;avg}$ or $v_{avg}$, it is used only as a supplementary comparison together with the crack growth curves, dislocation analysis, and hydrogen distribution results.

Based on this normalized comparison, the 15% Ni model shows the highest index ($I_{3}$ = 10.86) among the four studied compositions. This result is consistent with the crack propagation curve, where the 15% Ni model exhibits a clear plateau during the second stage and the shortest final crack length. It is also supported by the dislocation analysis. The moderate stacking fault energy in the 15% Ni model promotes sustained dislocation multiplication, cross-slip, and entanglement near the crack tip. These dislocation structures may help dissipate local stress and reduce apparent hydrogen mobility near the crack tip. Therefore, the normalized comparison further supports that under the present simulation conditions, the 15% Ni model showed the lowest crack propagation tendency among the investigated models.

### 4.5. Practical Implications and Limitations

The present results provide atomic-scale insight into hydrogen-resistant Fe-Ni alloy design based on idealized FCC Fe-Ni model systems. The comparison among different Ni contents suggests that hydrogen-assisted crack propagation is not controlled by Ni content alone. It is closely related to the combined effect of stacking fault energy, dislocation activity, and hydrogen mobility near the crack tip. Under the present simulation conditions, the 15% Ni model shows a more favorable response than the other studied compositions. Its intermediate stacking fault energy promotes sustained dislocation multiplication and entanglement near the crack tip, which may help dissipate local stress and restrict hydrogen migration. This result suggests that Ni content should be optimized to obtain a suitable stacking fault energy range, rather than simply increased. This design idea may be useful for Fe-Ni alloy systems used in hydrogen-containing environments.

However, several limitations should be noted. All compositions were constructed from an ideal FCC lattice, so the low-Ni models, especially A5 and A10, should be interpreted as idealized FCC Fe-Ni model systems. The simulation cell is at the nanoscale, the simulation time is limited to the picosecond scale, and the applied strain rate is much higher than that in real service. Therefore, the absolute values of the crack growth rate and apparent hydrogen mobility coefficient should not be directly used as engineering design parameters. In addition, the hydrogen concentrations of 5.3 at.% and 14.3 at.% represent local crack-tip enrichment rather than bulk hydrogen concentrations in engineering alloys, and each condition was simulated using one random distribution of Ni and H atoms. The present results should therefore be understood as comparative atomic-scale trends under selected configurations, not statistically averaged predictions. Future work should include larger-scale simulations, different random configurations, continuum fracture models, and experimental tests to improve the transferability of the present results.

## 5. Conclusions

This study established the mechanistic links among Ni content, stacking fault energy, dislocation behavior, hydrogen–defect interactions, and crack evolution in FCC Fe-Ni alloys using atomic-scale simulations. The local introduction of hydrogen at the crack tip had a limited effect on the overall macroscopic mechanical response of the model, with the peak stress of the A10 model remaining close to 7.35 GPa. However, hydrogen strongly affected crack propagation kinetics. During the second stage of crack growth, the crack propagation rate of the A10-14.3 at.% H model reached 0.36 Å/ps, which was much higher than that of the hydrogen-free and low-hydrogen models.

For all Ni contents, crack propagation showed three stages: initial slow propagation, rapid propagation, and later stable propagation. Among the studied models, the A15 model showed a clear crack propagation plateau in the second stage and had the shortest final crack length, indicating better resistance to hydrogen-assisted crack propagation.

Ni content controlled the dislocation evolution mode by regulating the stacking fault energy. The calculated SFE varied with Ni content and affected the dislocation evolution mode near the crack tip. The A5 and A20 models had relatively high SFE values and showed limited sustained dislocation retention near the crack tip. The A10 model had the lowest SFE, but its dislocation activity was still intermittent. By comparison, the A15 model showed a more favorable balance between dislocation mobility and dislocation accumulation, leading to sustained dislocation multiplication and entanglement during the second stage of crack propagation.

Hydrogen diffusion behavior was also closely related to Ni content and dislocation evolution. The second-stage apparent hydrogen mobility coefficient decreased from 5.86 Å^2^/ps in the A10-14.3 at.% H model to 4.31 Å^2^/ps in the A15-14.3 at.% H model. This indicates that hydrogen atoms were more easily trapped by dislocations, and their diffusion was restricted by dislocation binding. This weakened the continuous driving force for crack propagation. A supplementary normalized comparison based on the apparent hydrogen mobility coefficient and middle-stage crack propagation rate further confirmed that the 15% Ni model had the lowest crack propagation tendency among the investigated models, under the present simulation conditions. This work provides an atomic-scale theoretical basis for the composition design and microstructure control of alloys used in high-hydrogen service environments.

## Figures and Tables

**Table 1:** Relative crack growth rates for the 10% Ni crack model with different crack tip hydrogen concentrations.

Ni Content	Hydrogen Concentration	Stage I (0–60 ps) Relative Crack Propagation Rate (Å/ps)	Stage II (60–110 ps) Relative Crack Propagation Rate (Å/ps)	Stage III (110–200 ps) Relative Crack Propagation Rate (Å/ps)
10%	0	0.043	0.049	0.22
	5.3 at.%	0.045	0.026	0.22
	14.3 at.%	0.049	0.36	0.17

**Table 2:** The apparent hydrogen mobility coefficients for different models.

Model	Diffusion Coefficient (Å^2^/ps) of Stage I (0–60 ps)	Diffusion Coefficient (Å^2^/ps) of Stage II (60–120 ps)
A10-5.3 at.%H	0.037	4.61
A10-14.3 at.%H	0.2	5.86
A15-14.3 at.%H	0.21	4.31

## Data Availability

The original contributions presented in this study are included in the article. Further inquiries can be directed to the corresponding authors.
